# The risks of liver injury in COVID-19 patients and pharmacological management to reduce or prevent the damage induced

**DOI:** 10.1186/s43066-021-00082-y

**Published:** 2021-01-27

**Authors:** Antonio Vitiello, Raffaele La Porta, Vilma D’Aiuto, Francesco Ferrara

**Affiliations:** 1 Pharmaceutical Department, Usl Umbria 1, Via XIV settembre, 06132 Perugia, Italy; 2 Pathology Department, ASUR Marche, Urbino, Italy; 3 Pharmaceutical Department, Usl Umbria 1, A.Migliorati street, 06132 Perugia, Italy

**Keywords:** Liver fibrosis, Liver damage, COVID-19-SARS-CoV-2, Obeticholic acid

## Abstract

**Background:**

The global pandemic COVID-19 caused by the new coronavirus SARS-CoV-2 has already caused about 1.4 million deaths, and to date, there are no effective or direct antiviral vaccines. Some vaccines are in the last stages of testing. Overall mortality rates vary between countries, for example, from a minimum of 0.05% in Singapore to a maximum of 9.75 in Mexico; however, mortality and severity of COVID-19 are higher in the elderly and in those with comorbidities already present such as diabetes, hypertension, and heart disease.

**Main text:**

Recent evidence has shown that an underlying liver disease can also be a risk factor, and SARS-CoV-2 itself can cause direct or indirect damage to liver tissue through multisystem inflammation generated especially in the more severe stages. In the current pandemic, liver dysfunction has been observed in 14–53% of patients with severe COVID-19. In addition, drugs administered during infection may be an additional factor of liver damage. The mechanism of cellular penetration of the virus that occurs by viral entry is through the receptors of the angiotensin 2 conversion enzyme (ACE-2) host that are abundantly present in type II pneumocytes, heart cells, but also liver cholangiocytes.

**Conclusion:**

In this manuscript, we describe the clinical management aimed at preserving the liver or reducing the damage caused by COVID-19 and anti-COVID-19 drug treatments.

## Background

**Fig. 1:**
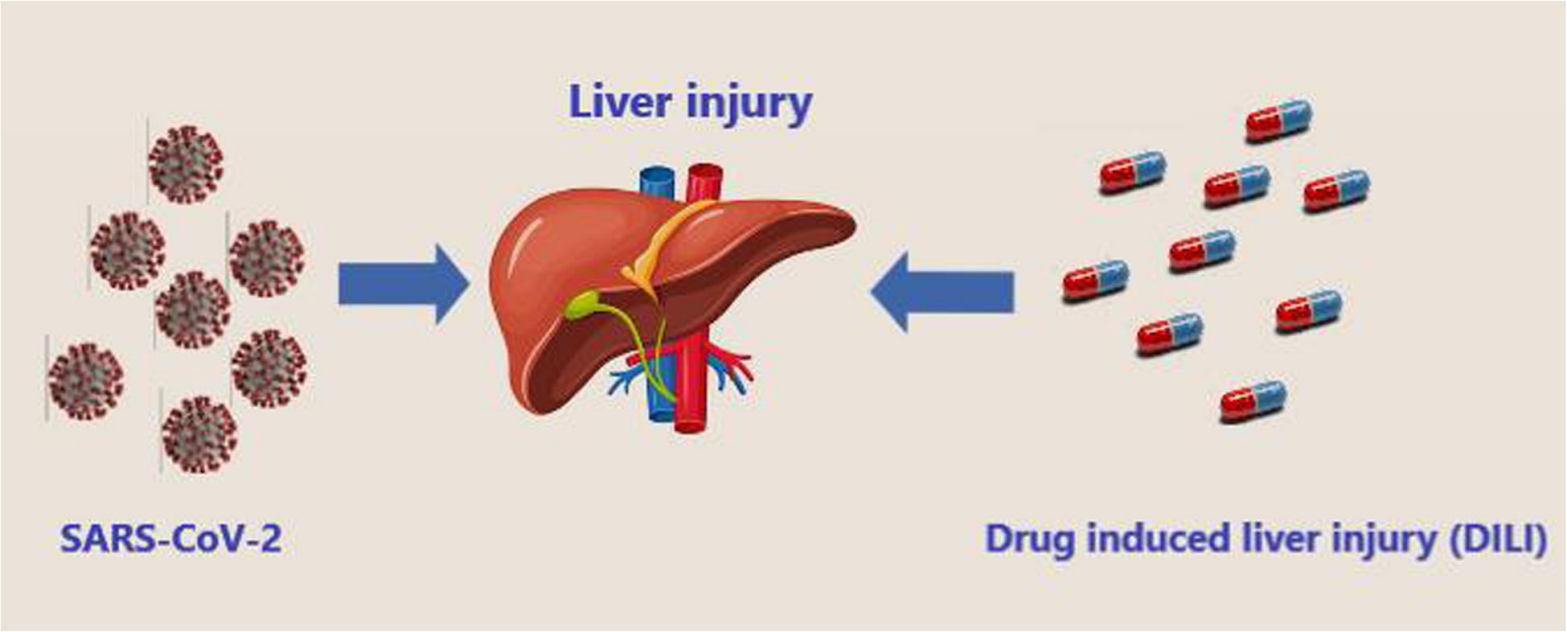
COVID-19 can cause direct liver damage, either through ACE-2 entry or indirect through generalized inflammation caused by the cytokine cascade. In addition, the administration of drugs with DILI potential may be an additional cause of liver damage

The new coronavirus SARS-CoV-2 (COVID-19) is the cause of severe acute respiratory syndrome (SARS), a severe form of viral pneumonia. The virus spread rapidly from China to the rest of the world in a very short time and with considerable intensity and severity creating a “global emergency.” The global pandemic COVID-19 caused by the new coronavirus SARS-CoV-2 has already caused about 1.4 million deaths [1]. SARS-CoV-2 is an RNA virus similar for about 80% of the viral genome to SARS-CoV (responsible for the 2003 outbreak) [2]. In vitro studies confirm that the virus penetrates human cells by binding to the protein ACE-2, the angiotensin 2 conversion enzyme, which is part of the renin-angiotensin system (RAS) [3] and is considered as a possible protein receptor. It is also known that patients infected with this virus, during sick days, show changes and variations in the concentrations of the enzyme components of RAS [4–6]. SARS-CoV-2 infection may also have a totally asymptomatic course. In a percentage of cases, however, the infection has a course consisting of an initial asymptomatic or slightly symptomatic phase and subsequent phases characterized by a generalized inflammatory state that causes multi-organ tissue lesions and respiratory distress syndrome [7]. According to observational studies conducted, most patients considered as severe cases present bilateral interstitial pneumonia and a hyperactive inflammatory state that is not only localized in lung tissue, but in all tissues of the body, causing multi-organ dysfunction and risk of thrombosis [8, 9]. The presence of generalized inflammation in all organs and the consequent increased risk of thrombosis require timely anti-inflammatory/immunomodulatory and anticoagulant treatment. The generalized inflammatory state responsible for serious injury is caused by an overactivation of the components of the host’s inflammatory/immune system characterized by a sudden high release of cytokines, an event called cytokine storm that leads to severe and sometimes fatal tissue damage. The mortality and severity of COVID-19 are higher in the elderly and in subjects with already present comorbidities such as diabetes, hypertension, and heart disease. Recent evidence has shown that even an underlying liver disease can be a risk factor, and SARS-CoV-2 itself can cause direct or indirect damage to liver tissue through multisystem inflammation generated especially in the most severe stages. Finally, medication intake during the period of infection can in some cases further aggravate the liver. In this context, it is essential to fight the virus but also to maintain the integrity of the organs with appropriate and targeted therapies. The angiotensin 2 conversion enzyme (ACE-2) is the receptor pathway of intracellular penetration of SARS-CoV-2. ACE-2 is expressed in many tissues such as the lungs, testicles, heart, and liver [10]. Studies have shown that the concentration of ACE-2 varies in the different stages of infection, particularly in the most severe phase a decrease has been shown [10]. Some evidence has shown that the receptor of the ACE-2 cell surface is expressed in liver tissue, more highly expressed in cholangiocytes (59.7%) than hepatocytes (2.6%). In particular, evidence shows that the level of expression of ACE-2 in cholangiocytes was similar to that of type 2 pneumocytes, indicating that the liver is a potential target organ for SARS-CoV-2. Instead, it seems that ACE-2 is poorly expressed by Kupffer cells [11]. In view of the above, the liver is a potential entry target for SARS-CoV-2. The involvement of liver damage from COVID-19 could be related to several factors. These include a direct damage of the virus penetrating by ACE-2 into the liver tissue, an uncontrolled inflammatory/immune reaction causing fibrosis and liver dysfunction, or a liver lesion caused by an anti-COVID-19 drug therapy [12] (Fig. 1).

In addition, considering all these factors, COVID-19 can cause a worsening of an underlying liver disease, causing liver decompensation resulting in increased mortality. Epidemiological studies indicate that 14–53% of COVID-19-positive patients have developed liver damage associated with a more negative outcome. Another study reports that cases of acute liver injury were reported in 13 (5%) out of 274 patients of whom 10 (76.9%) died [13]. Liver enzyme elevation has been observed mainly in severe COVID-19 patients, and in those on antiviral therapy, a higher percentage of liver enzyme elevation has been observed [14]. Also with previous similar epidemics, SARS and MERS, patients with liver enzyme elevation or with an already existing liver disease had a higher risk of serious complications [15, 16]. Ultimately, in addition to a possible direct liver damage caused by SARS-CoV-2, it is possible that the generalized and dysregulated inflammatory/immune state caused by a cytokine storm may be conducive to fibrosis and liver lesions. Further data are required to ascertain the pattern and degree of liver injury in patients with COVID-19. Finally, the pharmacological polytreatment to which a COVID-19 patient may be subjected may further aggravate the liver condition.

## Main text

### The risk of drug hepatotoxicity in COVID-19 patients

Drug-induced liver injury (DILI) is a liver lesion due to medication. Generally, the incidence is low but they are an important cause of acute liver failure with mortality or liver transplantation in emergency/urgency; the drugs able to cause it are many, and the diagnosis is difficult. DILI is a frequent cause of abnormal liver testing and an important cause of acute liver failure with mortality or liver transplantation. DILI is a frequent differential diagnosis in patients with acute liver injury. Patients admitted to hospital with COVID-19 positive may undergo pharmacological polytreatment making the clinical management even more complex [17]. In this context, information on the potential hepatotoxicity of the pharmacological agents in use is important in the diagnostic process, considering that the hepatotoxicity of drugs can vary depending on race, sex, and age [18]. There are many drugs that can affect liver function and damage it; some of them can cause elevation of liver enzymes asymptomatically; in other cases, acute hepatitis may occur; also liver damage may depend on the dosage of the drug used (e.g., paracetamol) or may be independent of drug dosage used. Among the drugs that can cause liver damage, there are drugs commonly used as antibiotics, anti-inflammatory, and antiviral [19]. A percentage of patients with COVID-19 may have an asymptomatic course of viral infection; a good percentage may have fever and use antipyretics such as paracetamol or other analgesics, with potential hepatotoxicity, which associated with the risk of liver damage that may occur in the most severe stages of COVID-19 infection and can result in a very dangerous synergy. Currently, there is no antiviral drug directed against SARS-CoV-2 and many patients with COVID-19 are administered antivirals authorized for other therapeutic indications such as remdesivir, lopinavir or ritonavir, and other drugs [20], with documented hepatotoxicity potential. In addition, there is evidence that the combination of lopinavir and ritonavir overdose can activate the endoplasmic reticulum stress pathway in the liver and induce hepatocyte apoptosis through the caspase cascade system and induce inflammatory reactions and oxidative stress by accelerating liver damage. The cause of liver enzyme elevations during ritonavir therapy is not fully known. Ritonavir is widely metabolized by the liver through the cytochrome P450 (CYP 3A4) system, which is also an inhibitor. Therefore, the production of a toxic intermediate of ritonavir or other agents metabolized by CPY3A4 may be the basis for potential liver injury [21, 22]. In the most severe stages of COVID-19 infection, a prothrombotic state may be responsible for the increased risk of thrombosis. The use of anticoagulants is a well-known cause of the potential risk of drug-induced liver damage (DILI) [23]. As described above, a generalized inflammatory state caused by a cascade of cytokines can cause multi-organ dysfunction and serious complications from COVID-19, with not only pulmonary but also cardiac or hepatic involvement. In patients with COVID-19, IL-6 inhibitors such as tocilizumab are used experimentally to reduce overactive inflammation. In patients treated with tocilizumab, episodes of severe drug-induced liver damage have been observed, including acute liver failure and hepatitis acute, which in some cases required a liver transplant [24]. Tocilizumab is known to cause a transient or intermittent increase in mild to moderate levels of liver transaminases, most frequently when used in combination with potentially hepatotoxic drugs. The mechanism by which it causes liver injury is unknown, but may be the result of its effects on the immune system or the IL-6 pathway that is important in liver regeneration. The incidence of liver damage caused by different drugs varies, but the incidence increases with the number of drugs administered. One of the serious complications that can occur during the course of COVID-19 is the formation of pulmonary fibrosis. Some studies are experimenting with the use of antifibrotic drugs such as pirfenidone [25]. Pirfenidone is an antifibrotic drug, commonly used in patients with idiopathic pulmonary fibrosis (IPF). Despite reports of reduced hepatic fibrosis, it is associated with the risk of hepatotoxicity. The mechanism by which pirfenidone may cause liver injury is unknown, but therapy with pirfenidone may be associated with mild to moderate serum aminotransferase elevations [26]. Experimentally, a treatment used in COVID-19 patients to reduce the inflammatory state is colchicine [27, 28]. This drug is also not free of DILI potential, although at low doses used it appears to have a good liver safety profile [29, 30]. In addition, liver dysfunction caused by COVID-19 may be responsible for incorrect liver metabolization of the drugs, with the risk of increased toxicity. The diagnosis of drug-induced liver lesions requires a combination of medical history and related tests to exclude other liver diseases and to assess the association between liver lesions and suspected causative drugs. An antiviral used in COVID-19 is the remdesivir [31, 32]. There are also reported cases of drug-related hepatotoxicity in patients with COVID-19 caused by the use of remdesivir, with likely interaction of P-glycoprotein (P-gp) inhibitors. Until further details on this interaction are available, it is recommended that patients be cautious with P-gp inhibitors in patients receiving remdesivir therapy [33].

### Pharmacological management

Liver damage can be responsible for serious complications in COVID-19 patients. In general, experience in the prevention and treatment of liver damage in patients in previous outbreaks caused by SARS-CoV may be a reference for the treatment of COVID-19 patients at risk of liver injury. For patients with COVID-19 and acute liver lesions, the causes of liver lesions should be analyzed and judged and appropriate measures should be taken, closely monitoring ALT, AST, total bilirubin, direct bilirubin, albumin, and PTA (INR) [34]. Patients with acute liver failure should be subjected to intensive monitoring and symptomatic and supportive treatment, and hypoproteinemia should be managed. In cases of drug-induced liver injury, in addition to conventional anti-inflammatory liver protection treatment, consideration should be given to changing the dosage or reducing the amount of suspected drugs, and the degree of liver damage should be assessed, followed by an adjustment of the treatment plan. Patients with severe liver damage caused by COVID-19 should be treated with hepatoprotective and anti-inflammatory agents [35]. However, pharmacological interactions with agents used against COVID-19 should be avoided. Patients with COVID-19 with a slight increase in liver enzymes generally do not need anti-inflammatory and hepatoprotective drugs. In patients with COVID-19, when administering antivirals or anti-inflammatory drugs, liver parameters should be monitored, and preventive application of drugs that protect the liver and reduce enzymes is not recommended. In patients with severe COVID-19, particularly with pre-existing liver diseases, too many drugs with DILI potential (generally no more than 2) should not be administered by carefully monitoring dosage and drug interactions, using appropriate dosages depending on renal and liver function. In the case of drug-induced liver damage, the suspect drug should be detected early and consideration should be given to discontinuing or reducing the dosage [36, 37]. In patients with ongoing anti-HBV or anti-HCV treatment, they should not discontinue therapy; however, careful monitoring of anti-COVID-19 therapies administered is necessary. The management of the reduction of the hyperactive and generalized inflammatory state should be implemented with appropriate therapies, considering that IL-1 or IL-6 drugs can be bifacial: on the one hand, they reduce inflammation; on the other hand, they have a DILI potential and worsen clinical conditions. However, control of the dysregulated inflammatory/immune state is “a must” to prevent the onset of systemic inflammatory response syndrome and reduce the likelihood of a mild disease developing into a severe or critical disease. In the presence of hepatic fibrotic tissue, even in the post-COVID-19 period, drug therapy could be considered to reduce the fibrotic and inflammatory state of the liver. In the pharmacological treatment of a disease, the safety aspects of medical therapy are important [38]. Recently, evidence has shown that obeticholic acid is showing excellent results for the treatment of non-alcoholic steatohepatitis-related liver fibrosis (NASH), offering a potential future option for an increasingly common liver disease without good current therapies. Obeticholic acid is a selective and potent agonist for the farnesoid X receptor (FXR), a nuclear receptor expressed at high levels in the liver and intestine. FXR is considered a key regulator of the biliary acid pathway and the inflammatory, fibrotic, and metabolic process. However, the use of obeticholic acid in liver fibrosis caused by COVID-19 is not supported and demonstrated by clinical epidemiological scientific evidence, but is only a scientific hypothesis of pharmacological treatment. Finally, it has recently been demonstrated that glycyrrhizinic acid derivatives may also have antiviral activity against SARS-CoV-2. Glycyrrhizin was the preferred anti-inflammatory drug for protection against liver disease and has been used in clinical practice for many years. In case of hepatotoxic drug intoxication, the use of silymarin can be considered. The use of obeticholic acid, glycyrrhizin, and silymarin is currently not supported by epidemiological evidence in COVID-19 patients [39].

## Conclusion

COVID-19 is currently an ongoing global pandemic. Viral infection in the most severe cases not only causes lung, but also cardiac and hepatic complications, which can be responsible for serious complications and some cases of fatal outcomes. A percentage of severe cases of COVID-19 reported cases of acute liver injury associated with higher mortality. More in-depth studies with long-term follow-up are needed to characterize the extent and cause of liver damage in COVID-19 and the clinical implications it has. In addition, a severe COVID-19 patient is a complex patient who may be subjected to pharmacological polypharmacy, with increased risk of liver damage. The effects of COVID-19 infection, and anti-COVID-19 treatments, on liver function require detailed and thorough evaluation, with further quality research in this area.

## Data Availability

Full availability of all data in the literature.

## Abbreviations

SARS-CoV-2/COVID-19/MERS: Coronavirus
ACE2: Angiotensin 2 conversion enzyme
RAS: Renin-angiotensin system
